# Management of Caesarean Scar Keloid in Pregnancy: A Multidisciplinary Approach

**DOI:** 10.7759/cureus.95375

**Published:** 2025-10-25

**Authors:** Abinaya Talluri, Venkata Sirisha Gurram

**Affiliations:** 1 Obstetrics and Gynaecology, Frimley Health National Health Service (NHS) Foundation Trust, Frimley, GBR; 2 Obstetrics and Gynaecology, Mid and South Essex National Health Service (NHS) Foundation Trust, Basildon, GBR

**Keywords:** caesarean section, intralesional triamcinolone, keloid, multidisciplinary care, pregnancy, scar management, wound healing

## Abstract

Keloid formation following caesarean section can be a distressing complication. Women of African, Hispanic or Asian descent are more predisposed to abnormal scar healing. During pregnancy, hormonal and mechanical factors may further aggravate keloid growth, leading to pain, cosmetic disfigurement, and emotional distress. Managing such cases requires careful coordination between obstetric, surgical, and dermatological teams to achieve safe maternal and cosmetic outcomes.

A 29-year-old gravida 2 para 1 woman of African origin, with a previous history of emergency caesarean section, was seen at 27+4 weeks of gestation following transfer of care. Examination revealed an extensive keloid extending from the lower abdomen to the vulval region. The pregnancy remained otherwise uncomplicated. After multidisciplinary discussion, an elective caesarean section with simultaneous keloid excision was planned. The patient underwent elective term caesarean delivery with complete excision of the abdominal and vulval keloids, using an incision along the inner edge of the keloid and deep dissection. The wound was closed with tension-free subcuticular sutures, and intralesional triamcinolone was administered to minimise recurrence. Given that recurrence rates following excision alone can reach 45-100%, adjuvant corticosteroid use was chosen to reduce the risk to below 20%. The postoperative period was uneventful, and wound healing was satisfactory with no recurrence of keloid formation.

This case highlights the importance of a multidisciplinary, individualised approach in managing extensive keloids during pregnancy. Combining meticulous surgical technique with corticosteroid therapy can result in good functional and cosmetic outcomes. Early identification and involvement of specialist teams are essential to optimise care for women at higher risk of keloid formation.

## Introduction

Keloids are benign fibroproliferative lesions that develop due to an exaggerated wound healing response. They are characterised by excessive collagen deposition beyond the original wound margins, resulting in raised, firm, and often symptomatic scars. Keloids occur in approximately 10-15% of individuals with darker skin types, particularly those of African, Asian, or Hispanic descent, owing to genetic and hormonal factors influencing fibroblast activity [1,2].

Pregnancy can exacerbate keloid development due to elevated levels of estrogen, progesterone, and growth factors that promote fibroblast proliferation and collagen synthesis. Keloids that form after caesarean section can cause significant discomfort, pruritus, cosmetic disfigurement, and psychological distress. Their management during pregnancy is complex and requires balancing maternal safety, aesthetic outcome, and recurrence prevention through a multidisciplinary approach [3].

Reported recurrence rates after surgical excision alone range from 45-100%; this risk can be reduced to 10-50% with adjunctive intralesional corticosteroid therapy and below 20% when combined with radiotherapy [4-6]. This report describes the management of extensive keloids in a pregnant woman with a previous caesarean section scar, illustrating the value of coordinated care between obstetrics and plastic surgery teams.

## Case presentation

**Figure 1:**
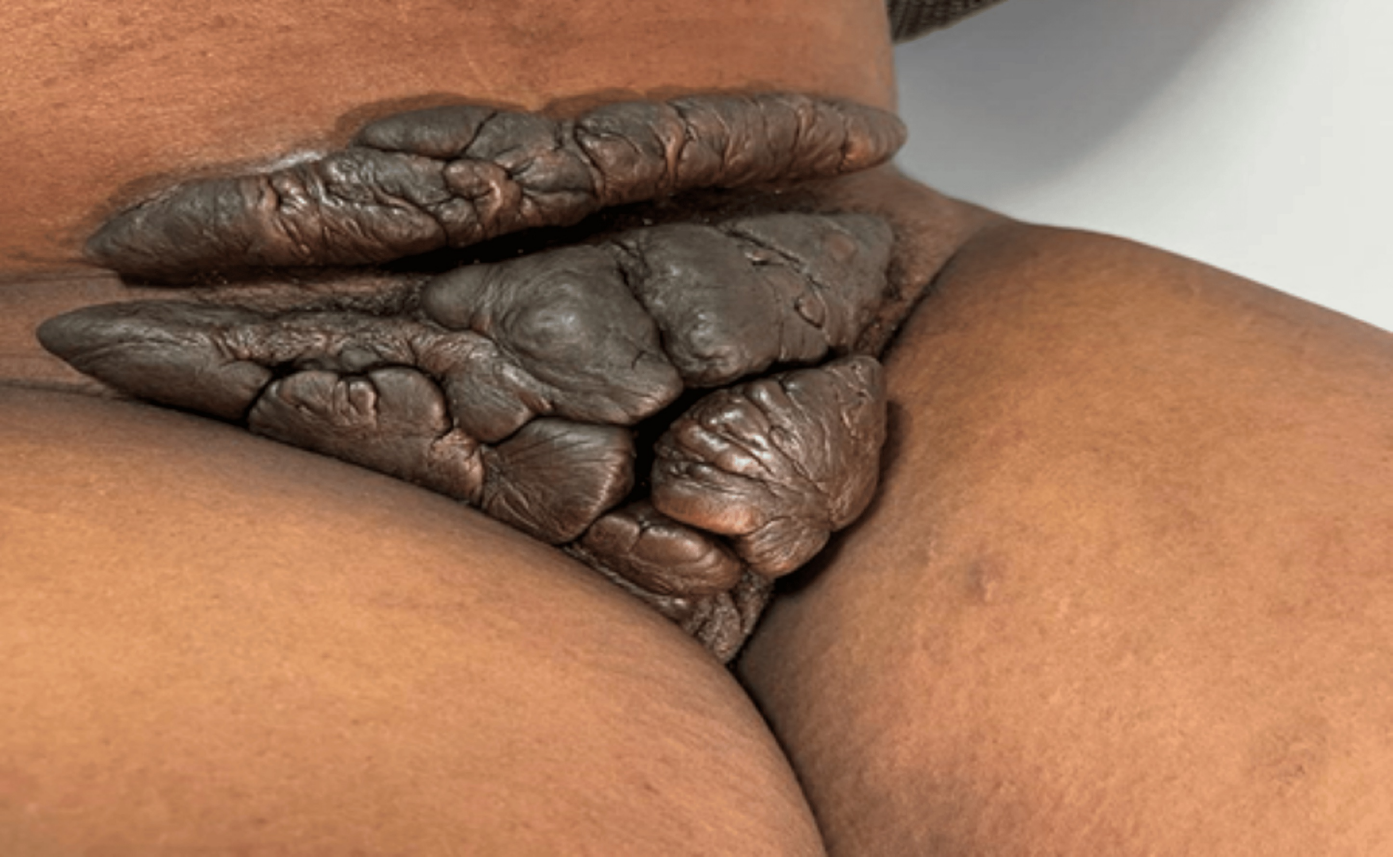
Preoperative patient image Preoperative examination showed an extensive keloid extending from the lower abdomen to the vulval region.

A 29-year-old gravida 2 para 1 woman of African origin presented at 27+4 weeks’ gestation following transfer of antenatal care. Her medical history included an emergency caesarean section in 2020 for failed induction at 41 weeks, performed abroad and prior keloid excision (on her back following an Injury) in 2017. She denied smoking, alcohol use, or other medical conditions. She was a late booker for antenatal care and reported no prior cervical screening. On examination at 32+1 weeks, she had a large keloid extending from the lower abdomen from her previous caesarean section and involving the vulval region, associated with discomfort and local irritation, as can be seen in Figure 1.

Speculum examination excluded active cervical pathology. Vaginal swabs were positive for Group B Streptococcus, for which appropriate intrapartum prophylaxis was planned. Given the extent of the lesions and the patient’s symptoms, she was referred to the vulval clinic and subsequently reviewed by a multidisciplinary team comprising obstetricians and plastic surgeons. A plan was made for an elective caesarean section with concurrent excision of the keloid at term. A multidisciplinary team (MDT) comprising obstetricians and plastic surgeons planned the procedure and the obstetricians executed the procedure.

**Figure 2:**
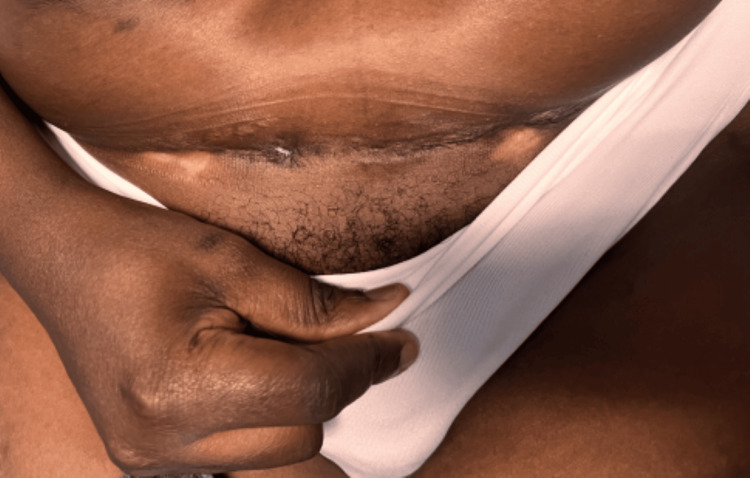
Postoperative image of the patient Postoperative appearance at six weeks demonstrated well-healed scars with no recurrence.

At the time of surgery, the caesarean incision was placed along the inner margin of the keloid to allow complete excision. Deep dissection was performed to remove the fibrotic tissue. The wound was closed in layers using tension-free subcuticular sutures with 3-0 Monocryl to minimise stress on healing tissues and prevent recurrence. Intralesional triamcinolone acetonide (40 mg/mL) was injected along the upper and lower wound edges to inhibit fibroblast proliferation and reduce inflammation. Prophylactic antibiotics were administered preoperatively in the first 24 hours to prevent infection. The postoperative recovery was uneventful. Wound healing was good, with no evidence of infection or early recurrence at six weeks, as shown in Figure 2.

Written informed consent was obtained from the patient for publication of this case report, including all clinical details and accompanying pre- and postoperative images.

## Discussion

Keloid formation represents a pathological response to dermal injury involving persistent inflammation and abnormal collagen turnover. Individuals of African descent are particularly predisposed due to genetic and immunological factors influencing fibroblast activity [4]. In pregnancy, elevated hormonal levels and increased skin tension contribute to keloid progression [3]. Caesarean scar keloids can significantly impact quality of life, leading to pain, pruritus, and cosmetic distress. Their management remains challenging, as recurrence is common even after surgical excision. Evidence supports a multimodal strategy combining surgery, corticosteroid injection, and radiotherapy to achieve optimal outcomes [5,6].

Recurrence rates after different treatment modalities are as follows: (a) surgical excision alone: 45-100% [6], (b) excision+intralesional triamcinolone: 10-50% [7], (c) excision+radiotherapy: <10-20% [8]. In our patient, we used a combination of surgical excision with deep dissection and tension-free closure, along with intralesional corticosteroids. Surgical excision provides immediate symptomatic relief but carries a high recurrence risk if performed in isolation. To minimise this, tension-free closure and the use of subcuticular sutures are recommended [7]. Adjunctive intralesional corticosteroid therapy, such as triamcinolone acetonide, reduces fibroblast proliferation and collagen synthesis, improving both recurrence and symptom control [8].

Radiotherapy within 24-48 hours post-excision has been shown to substantially reduce recurrence rates by inducing fibroblast apoptosis and modulating cytokine activity [9]. Fractionated low-dose radiotherapy is considered safe in the postpartum setting, with minimal radiation exposure to reproductive organs. There appears to be no gold standard for treating keloids or preventing recurrence. Emerging combinations such as laser-assisted steroid delivery, 5-fluorouracil plus corticosteroids, and autologous fat grafting show promising results [10,11].

Fewer than 10 published reports have described keloid management during pregnancy, most focusing on limited abdominal lesions. To our knowledge, this is among the first reports describing coordinated management of an extensive lower abdominal caesarean-scar keloid during pregnancy, highlighting the value of multidisciplinary planning at the time of delivery. The patient was advised to attend long-term follow-up; however, she has not re-presented since six weeks postpartum. However, recurrence may occur up to two years postoperatively. A long-term evaluation is planned to document the durability of results.

## Conclusions

Keloid formation following caesarean section can cause significant physical and psychological morbidity, particularly in women with darker skin types. Successful management requires early multidisciplinary involvement and an individualised treatment plan that integrates meticulous surgical technique, intralesional corticosteroid therapy, and, where appropriate, adjuvant radiotherapy. A combined approach not only improves functional and cosmetic outcomes but also helps minimise recurrence. Quantitative recurrence data have been incorporated to strengthen the evidence base for management and counselling. Attention to the psychosocial impact and provision of culturally sensitive counselling are equally important aspects of care. Early recognition, patient-centred planning, and collaboration between obstetric and plastic surgery teams are key to optimising results and supporting women at higher risk of keloid formation.
